# Bioanalytical Method Development and Validation Study of Neuroprotective Extract of Kashmiri Saffron Using Ultra-Fast Liquid Chromatography-Tandem Mass Spectrometry (UFLC-MS/MS): In Vivo Pharmacokinetics of Apocarotenoids and Carotenoids

**DOI:** 10.3390/molecules26061815

**Published:** 2021-03-23

**Authors:** Aboli Girme, Sandeep Pawar, Chetana Ghule, Sushant Shengule, Ganesh Saste, Arun Kumar Balasubramaniam, Amol Deshmukh, Lal Hingorani

**Affiliations:** Pharmanza Herbal Pvt. Ltd., Anand, Gujarat 388435, India; lcms@pharmanzaherbals.com (S.P.); adic@pharmanza.com (C.G.); rdbiotech@pharmanza.com (S.S.); ard@pharmanzaherbals.com (G.S.); process@pharmanza.com (A.K.B.); rd@pharmanzaherbals.com (A.D.); lal@pharmanzaherbals.com (L.H.)

**Keywords:** crocetin, crocin, picrocrocin, safranal, dietary supplement, nutraceutical

## Abstract

Kashmir saffron (*Crocus sativus* L.), also known as Indian saffron, is an important Asian medicinal plant with protective therapeutic applications in brain health. The main bioactive in Kashmir or Indian Saffron (KCS) and its extract (CSE) are apocarotenoids picrocrocin (PIC) and safranal (SAF) with carotenoids, crocetin esters (crocins), and crocetins. The ultra-fast liquid chromatography(UFLC)- photodiode array standardization confirmed the presence of biomarkers PIC, *trans*-4-GG-crocin (T4C), *trans*-3-Gg-crocin (T3C), *cis*-4-GG-crocin (C4C), *trans*-2-gg-crocin (T2C), *trans*-crocetin (TCT), and SAF in CSE. This study’s objectives were to develop and validate a sensitive and rapid UFLC-tandem mass spectrometry method for PIC and SAF along T4C and TCT in rat plasma with internal standards (IS). The calibration curves were linear (*R*^2^ > 0.990), with the lower limit of quantification (LLOQ) as 10 ng/mL. The UFLC-MS/MS assay-based precision (RSD, <15%) and accuracy (RE, −11.03–9.96) on analytical quality control (QC) levels were well within the acceptance criteria with excellent recoveries (91.18–106.86%) in plasma samples. The method was applied to investigate the in vivo pharmacokinetic parameters after oral administration of 40 mg/kg CSE in the rats (*n =* 6). The active metabolite TCT and T4C, PIC, SAF were quantified for the first time with T3C, C4C, T2C by this validated bioanalytical method, which will be useful for preclinical/clinical trials of CSE as a potential neuroprotective dietary supplement.

## 1. Introduction

*Crocus sativus* L. (CS), also known as saffron, is a popular food condiment. Cultivation of CS occurs in many countries, including Iran, India, Italy, Spain, and Greece. The flower stigmas of saffron have usage worldwide for traditional and medicinal use. This saffron mainly grows in India’s Kashmir region, known as Kashmir or Indian Saffron (KCS).

**Figure 1:**
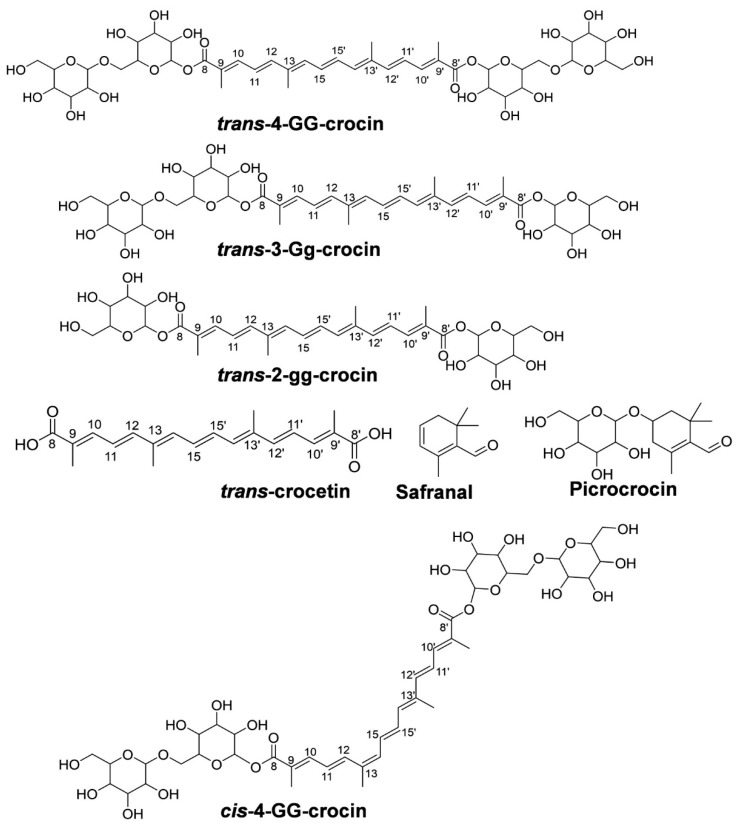
Compounds of Kashmir saffron extract (CSE)-*trans*-4-gg-crocin (T4C); *trans*-3-Gg-crocin (T3C); *trans*-2gg-crocin (T2C); *trans*-crocetin (TCT); safranal (SAF); picrocrocin (PIC), and *cis*-4GG-crocin (C4C).

This KCS is rich in bioactive constituents, carotenoids (crocetin esters, i.e., crocins, crocetin), and apocarotenoids such as picrocrocin (PIC) with safranal (SAF) (Figure 1) [1,2,3,4]. The Srinagar, Pulwama, Kishwar, and Badgam districts have been geographically and climatically suitable for the growth and enrichment of bioactive compounds in saffron [3,4,5]. Geographical indicators (GIs) for the production of Kashmir saffron have been validated [5]. It shows the growth and scope of production and the consistent supply of KCS with agricultural benefit.

Saffron’s stigma mainly contains the water-soluble carotenoids crocetins, and its esters, known as crocins responsible for this spice’s coloring properties. The esterification of crocetins with sugars like neapolitanose, gentiobiose (G), and glucose (g) gives the geometric isomers of crocins, where *trans* isomers dominate *cis* form [3,4]. The general crocins content ranges between 16–28% in the CS, and in some harvest years, it can represent up to 30% [4,6,7]. The apocarotenoid PIC is mainly responsible for the taste of CS, which is 7–16% in the dried stigmas of CS and can reach up to 20% [6,8,9,10]. Safranal is the bioactive precursor form of PIC which contributes to its aroma and is usually found as 0.1–0.6% in concentration in the CS on a dried weight basis (*w*/*w*) [6,11,12,13,14,15,16,17].

Saffron and their associated carotenoid–apocarotenoids were extensively studied over the last decade for their biomedical properties, especially for their brain health and chemopreventive potential. The compounds have found a highly antioxidant effect in neurodegenerative diseases with its relevance to Alzheimer’s (AD) and Parkinson’s Disease [3,18,19,20,21].

Kashmir or Indian Saffron holds importance in culinary and remedial values in India’s regional and traditional medicines. It has curative properties in nervous system functions and insomnia, depression, cataracts, night blindness, low vision while normalizing heart functions. Kashmir or Indian Saffron is traditionally used in India as antispasmodic and aphrodisiac, treating dermal diseases, kidney–urinary disorders, and rheumatoid arthritis with widespread cosmetics usage as per folklore, Ayurveda, Siddha, Sowa Rigpa, and Unani medicinal systems [1,22,23,24,25,26,27]. The recent scientific studies on KCS have shown its potential in AD, cardiac functions, immunomodulatory activity, and anti-tumor agents [3,28,29,30,31,32,33,34]. This increased valuation and consistent growth in the KCS consumption in health benefits have prompted the demand and interest in research as a dietary supplement and nutraceutical.

The data for bioanalytical studies showed that HPLC-based quantification had been reported for the presence of *trans*-4-GG-crocin (T4C) or crocin-1 and *trans*-crocetin (TCT) in CS [4,35,36,37]. But there is no report of the methodology for bioactive apocarotenoids PIC and SAF along with other carotenoids *trans*-3-Gg-crocin (T3C), *trans*-2-gg-crocin (T2C), *cis*-4-GG-crocin (C4C) from CS [35,36,37]. This research also lacks confirmation and detection of these compounds in biological samples after CS or its extract or product consumption. There is a need to develop a short and adaptable LC-based method with MS/MS to identify and quantify these compounds in a validated way. Current bioanalytical studies lack validation data and sensitivity for the bioactive compounds T2C, T3C, C4C, SAF, and PIC from CS as per the literature [4,38,39,40,41].

In the previous studies, the neuroprotective effect of KCS extract has been evaluated. An integral approach to prevent or delay AD is the blood-brain barrier (BBB) integrity and amyloid β-protein (Aβ) clearance. In an in vitro study, KCS extract enhanced the tightness of bEnd-3 cells-based BBB in the concentration of 0.2–0.22 mg/mL, thereby increasing Aβ clearance which helps in AD condition. This KCS extract has upregulated synaptic proteins and reduced neuroinflammation associated with Aβ pathology in 5XFAD (AD model) mice brains. The treatment of active metabolite crocetin resulted in improved memory function by inducing autophagy mediated by AMPK pathway activation in mice in a separate study, which states the study requirement for in vivo therapeutics data [3,4,42].

The UFLC-PDA standardization showed bioactive compounds PIC, T4C, T3C, C4C, T2C, TCT, and SAF in the KCS extract (CSE). Therefore, the present study aimed to develop and validate a sensitive and short UFLC-MS/MS bioanalytical method for the simultaneous determination of apocarotenoids (PIC, SAF) and carotenoids (T4C, TCT) in the rat plasma. This methodology was further validated with internal standards reserpine and chloramphenicol as per the USFDA (United States Food and Drug Administration)and EMA(European Medicines Agency guidance for linearity, precision, accuracy, recovery, extraction efficiency, stability, dilution integrity, and matrix effect. [40,41] This novel method was utilized for in vivo oral pharmacokinetics investigations of CSE in rats. The PIC, SAF, T4C, TCT, T3C, C4C, T2C, were detected in rat plasma using this validated bioanalytical method. This method and results will be beneficial in the further investigation of KCS and CSE. According to the phytopharmaceutical guidelines of the Drugs Controller General (DCG) (India) and other regulations, this bioanalytical method will be useful for preclinical or phase 1 clinical trials.

## 2. Results

The quantitation results of CSE (*n =* 3) with the concentrations of PIC, SAF, T4C, T3C, C4C, T2C with TCT was found as shown in Table 1.

### 2.1. Optimization of Chromatographic Conditions

The ultra-fast liquid chromatography-tandem mass spectrometry (UFLC-MS/MS) methodology was optimized by selecting the mobile phase system and promoting the retention of targeted analytes in pre-column and cleaning interferences. The water and acetonitrile (ACN) (80% to 10%) containing formic acid (0.01% to 0.1%) were compared, and water (0.1% formic acid) with ACN was selected as a mobile phase. Different mobile phase additives were tested systematically in the mobile phase optimization, including ammonium formate, ammonium acetate, and formic acid in ascending concentrations from 0 to 10 mmol/L and 0.01% to 0.1% to enhance the sensitivity and resolution of target compounds.

The mobile phase flow rate was optimized to increase the method’s separation and reproducibility as 0.8 mL/min after various trials of 0.4, 0.5, 0.6, and 0.7 mL/min flow rates (system pressure of UFLC < 4000 psi). The analyte transfer’s ideal time entirely from precolumn to the analytical column was optimized by switching at times 0, 0.5, 1 min, and 0.0 min. It was found that the response was proportional to the injection volume; hence optimization of injection volume was done with 5, 10, 20, and 50 µL. An increase in system pressure observed at 50 µL injection volume when compared to the rest. The lower limit of quantification (LLOQ) decreased ten-fold by changing the injection volume of 20 µL to 5 µL. Theoretically, the sample’s sensitivity is directly proportional to the injection volume loaded into the analytical column. Therefore, the column conditions with optimized injection volume influence the retention process, efficiency, peak shape, which found 20 µL. Under the optimized conditions, the retention times (tR) of T4C, T3C, T2C, C4C, TCT, PIC, SAF, reserpine (IS1), and chloramphenicol (IS2) were found as 3.90, 4.12, 4.74, 4.53, 5.96, 1.93, 6.19, 4.82 and 4.42 min, respectively. (Table 2).

### 2.2. Optimization of Mass Spectrometric Conditions

The optimization of MS conditions was performed on precursors and product ions of the analytes and IS. The electrospray ionization (ESI) interface was selected as T4C, T3C, T2C, C4C, and TCT with IS2 in a negative mode and SAF, PIC, with IS1 in a positive mode for a maximum and stable response. The mass parameters such as Q1 pre-bias and (DP), Q3 pre-bias, and collision energy values for all the analytes and IS1 and IS2 were optimized and shown in Table 2.

Carotenoids are fragile and naturally occurring isomerized compounds in CSE. The T4C and C4C are isomers with four sugar moieties at a molecular weight of 976.38 g/mol. Similarly, T3C is a carotenoid with three sugar moieties (814.33 g/mol) and T2C with two sugar moieties (652.27 g/mol). The basic chemical skeleton of these molecules is TCT with 328.17 g/mol. Similarly, the main apocarotenoid, PIC, has a molecular weight of 330.17 g/mol with a similar SAF structure, with a molecular weight of 150.10 g/mol with the sugar moiety.

**Figure 2:**
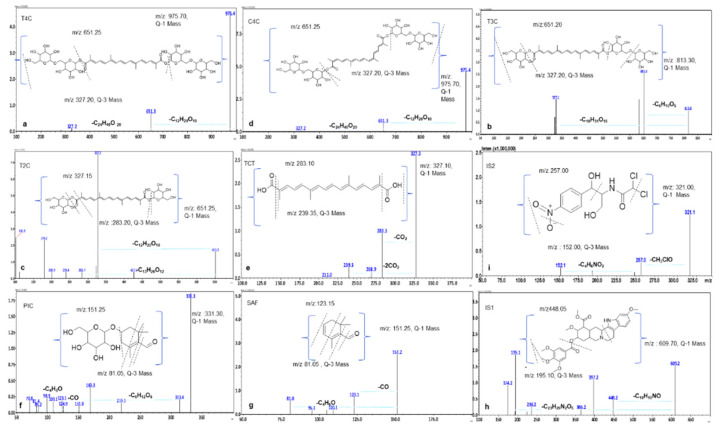
Possible mass fragmentation pattern and (*m/z*) values of precursor (Q1) and product (Q3) ions for T4C, T3C, T2C, C4C, TCT, IS2 (negative mode) and PIC, SAF, IS1 (positive mode) in the optimized ultra-fast liquid chromatography-tandem mass spectrometry (UFLC-MS/MS) based MRM conditions.

**Figure 3:**
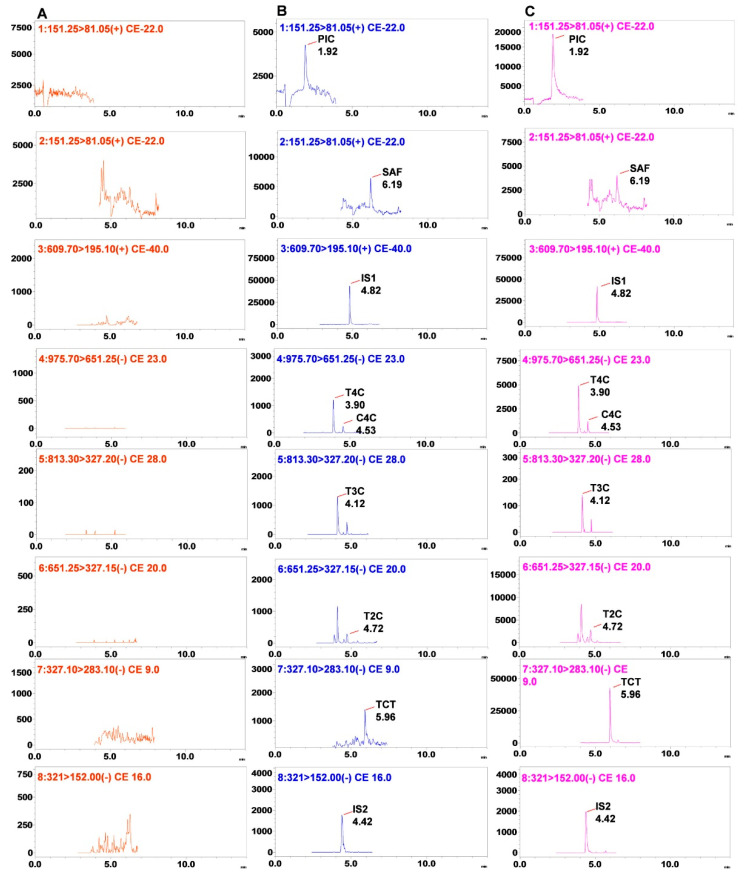
Typical MRM chromatograms of the seven components in rats: (**A**) blank plasma; (**B**) blank plasma sample spiked with standard mixtures and internal standard; (**C**) rat plasma samples collected after oral administration of the CSE. PIC, T4C, T3C, C4C, T2C, reserpine (IS1), TCT, SAF, chloramphenicol (IS2).

The Q3 MS spectra stabilized for all these compounds T4C, T3C, C4C, T2C, TCT, PIC, and SAF in this method. The spectra of product ions (qualifier and quantifier) showed transitions for T4C, T3C, C4C at 651.25/327.20, for T2C 327.15/283.20. The TCT showed a major transition at 283.10/239.35. In comparison, apocarotenoids PIC and SAF showed transition at 123.15/81.05 and 123.15/81.05, whereas 448.05/195.10 and 257.00/152.00 were used for IS1 and IS2 in multiple reaction monitoring (MRM) scan mode. (Figure 2 and Figure 3)

#### 2.2.1. Carotenoids–Apocarotenoids: Mass Fragmentation Analysis by UFLC-MS/MS

The precursor and fragmented ions from UFLC-MS/MS methodology were further studied for all carotenoids and apocarotenoid’s possible mass fragmentation pattern, as shown in Figure 2.

The ESI-MS (negative) spectrum of T4C (tR = 3.90 min) displayed a molecular ion at *m/z* 975.70 [M–H]^−^ and other two diagnostic peaks were observed at *m/z* 651.25 [M–H–C_12_H_20_O_10_]^−^ and *m*/*z* 327.20 [M–H–C_24_H_40_O_20_]^−^, confirming the presence of four glucose moieties [43]. The deprotonated molecular ion peak of the ESI-MS (negative) spectrum of T3C (tR= 4.12 min) displayed an ion at *m/z* 813.30 [M–H]^−^. The other two additional diagnostic peaks were observed at *m/z* 651.20 [M–H–C_6_H_10_O_5_]^−^ and *m/z* 327.20 [M–H–C_18_H_31_O_15_]^−^ confirm the presence of three glucose moieties. The deprotonated molecular ion peak of ESI-MS (negative) spectrum of T2C (tR = 4.74 min) displayed an ion at *m/z* 651.25 [M–H]^−^ and another two additional diagnostic peaks were observed at *m/z* 327.15 [M–H–C_12_H_20_O_10_]^−^ and *m/z* 283.20 [M–H–C_13_H_20_O_12_]^−^ which confirm the presence of two glucose moieties.

The ESI-MS (negative) spectrum of C4C (tR= 4.53 min) displayed a molecular ion at *m/z* 975.70 [M–H]^−^ and two additional diagnostic peaks were observed at *m/z* 651.25 [M–H–C_12_H_20_O_10_]^−^ and *m/z* 327.20 [M–H–C_24_H_40_O_20_]^−^, which confirmed the presence of four glucose moieties. The deprotonated molecular ion peak of ESI-MS (negative) spectrum of TCT (tR= 5.96 min) displayed a molecular ion at *m/z* 327.10 [M–H]^−^ [44], and other two additional diagnostic peaks were observed at *m/z* 283.10 [M–H–CO_2_]^−^ and *m/z* 239.35 [M–H–2CO_2_]^−^.

The apocarotenoids are carotenoids derived compounds with isoprenoids units present in CS [45]. In MS/MS analysis, protonated molecular ion peak observed for PIC as *m/z* 151.25 [M+H]^+^ at tR=1.93 min, with additional signals as *m/z* 123.15 [M+H–CO]^+^ and *m/z* 81.05 [M+H–C_4_H_6_O]^+^. While for SAF, the molecular ion at *m/z* 151.25 [M+H]^+^, with additional signals at *m/z* 123.15 [M-H-CO]^+^ and *m/z* 81.05 [M+H–C_4_H_6_O]^+^, was observed at tR=6.19 min. Both compounds could be separately identified by analyzing reference standards and retention time.

The [M+H]^+^ spectrum of reserpine (IS1) (tR 4.82 min) displayed a molecular ion at *m/z* 609.70 [M+H]^+^, and two additional signals were observed at *m/z* 448.05 [M+H–C_10_H_10_NO]^+^, and *m/z* 195.10 [M+H–C_23_H_29_N_2_O_5_]^+^. The spectrum of chloramphenicol (IS2) (tR=4.42 min) displayed a molecular ion at *m/z* 321.00 [M–H]^−^ and two additional signals were observed at *m/z* 152.00 [M–H–C_4_H_6_NO_2_]^−^ and *m/z* 257.00 [M–H–CH_3_OCl] (Figure 2). This data gives us possible ion fragmentation and application of this methodology in the proposed MS-based fragmentation pattern of these analytes from CSE.

#### 2.2.2. Linearity and Lower Limit of Quantification (LLOQ)

The calibration curves, correlation coefficients (*R*^2^), linear ranges, and LLOQ of the analytes are shown in Table 3. The linearity of the calibration curves was determined by plotting the peak area ratio (analytes/IS) against the nominal concentration of the calibration standards in rat plasma covering the expected range (10–3200 ng/mL) by linear regression analysis with the use of a 1/X^2^ (x is the concentration) weighing factor. The calibration curves of the analytes exhibited linearity at a specific concentration range in rat plasma.

The LOD was determined on the analyte concentration, which showed a peak response with a signal-to-noise ratio (S/N) of >3.3. Further, the LLOQ was determined with a signal-to-noise ratio > 10 at 10 ng/mL level (*n* = 5) for each analyte [40,41], which indicated that the method was sufficiently sensitive for pharmacokinetic studies. It is the lowest concentration of the standard curve that can be accurately and precisely measured.

### 2.3. Bioanalytical Method Validation

The following validation results were recorded and analyzed for four bioactive compounds (i.e., TCT, T4C, PIC) and SAF by UFLC-MS/MS in rat plasma.2.3.1. Optimization of Analytes Extraction from the Plasma

#### 2.3.1. Optimization of Analytes Extraction from the Plasma

While processing the rat plasma samples, analytes extraction is a critical step for accurate and consistent MS/MS analysis. Protein precipitation extraction (PPE) and liquid-liquid extraction (LLE) methods were compared for sample preparation. The carotenoids and apocarotenoids classes are the least stable in natural products. In CSE, the polar solvents are the solvent of choice for better solubility and consistent extraction. After different trials, acetonitrile and methanol were found as efficient solvents. Methanol was found to have better precipitation and stability of the analytes and IS experiments for CSE and was chosen as the optimized solvent for experimentation.

The simple and stable protein precipitation (PPE) method was optimized for the CSE analyte and internal standard extraction. The accuracy and matrix effect achieved in the PPE method using acidified methanol with formic acid also enhanced recovery and efficiency. This extraction method was suitable for stabilizing SAF content in the rat plasma compared to other trials. The quantification was done by using a response factor of IS. This concentration-dependent strategy was found helpful for CSE carotenoids–apocarotenoids for encountering variable responses.

#### 2.3.2. System Suitability Test

The system suitability test was applied to each batch at the LQC level (*n =* 5), during and after the batch analysis. The relative standard deviation of peak area response and retention time for each analyte was ≤10% and 5%, respectively.

#### 2.3.3. Specificity and Sensitivity

Validation was carried out as per USFDA and EMA guidelines [40,41]. The specificity and selectivity were investigated by comparing the retention times in chromatograms of blank plasma added with analytes and blank plasma, and plasma sample after oral administration of CSE; no interference peak was observed at a retention time of analytes of endogenous substances in the plasma samples. (Figure 3) The mean signal-to-noise ratios for T4C, TCT, PIC, SAF, IS1, and IS2 were 18.90 ± 4.28, 24.97 ± 4.82, 17.02 ± 3.39, 34.70 ± 7.08, 77.72 ± 8.28, 88.69 ± 10.77, respectively.

#### 2.3.4. Quality Control Samples

The accuracy and precision parameters determined on the four QC levels and other parameters and assay were calculated with three QC levels based on linearity and LLOQ [41] (Table 3). The QC samples were prepared at four levels by spiking the analyte in plasma to get the LLQC (10 ng/mL), LQC (three times of LLOQ, 30 ng/mL), MQC (50% of the upper CC range, 1600 ng/mL), and HQC (75% of the upper CC range, 2400 ng/mL).

#### 2.3.5. Accuracy and Precision

Precision and accuracy were determined by analyzing QC samples at four level concentrations (*n =* 6) on the same days (01–04) and a different day (*n =* 6 × 3). The accuracy was evaluated by relative error (RE). In contrast, precision was expressed in relative standard deviation (RSD), as given in Table 4. The range of precision from 1.29 to 10.18% for intra-day ranged from 1.16 to 10.51% for inter-day, respectively. The accuracy expressed was within ±15% in all QC levels. All the results met the acceptance criteria. This data indicated that this developed UFLC-MS/MS method was accurate and reliable for determining compounds.

#### 2.3.6. Extraction Recovery, Dilution Integrity, and Carryover

The results of the extraction recoveries and dilution integrity of the four compounds are listed in Table 5 and Table 6. The mean extraction recoveries of the analytes in plasma at three quality control (QC) concentration levels were found from 91.18 ± 0.73% to 106.86 ± 1.73%.

Dilution integrity determined by analyte spiking above the upper limit of quantification (ULOQ) and diluted with blank plasma brought into the calibration curve was analyzed to obtain acceptable concentration after proper dilution with blank plasma. The results showed that the precision and accuracy of the diluted QCs were within the 15% limit (Table 6).

#### 2.3.7. Matrix Effect and Stability Studies

The four compounds’ matrix effects were found to be in the range of 95.88± 8.44% to 100.78 ± 2.59%, suggesting no significant ion suppression or enhancement in this LC-MS method as the guidelines in Table 7.

The analyte stability in plasma was demonstrated by analyzing LQC and HQC samples (*n =* 6) at storage conditions as per Table 8 for processed plasma samples of CSE. It showed that T4C, TCT, PIC, and SAF were stable but under restricted storage conditions due to their sensitive nature. The precision was found for autosampler stability conditions from approximately 3.08% to 4.76% with accuracy (RE, −0.19–3.40), for room temperature from 2.38% to 5.35% with accuracy (RE, −0.26–1.33), and freeze-thaw stability 2.43% to 8.25% with accuracy (RE, −0.06–4.13).

#### 2.3.8. Application of Bioanalytical Method to Oral Pharmacokinetics Study

**Figure 4:**
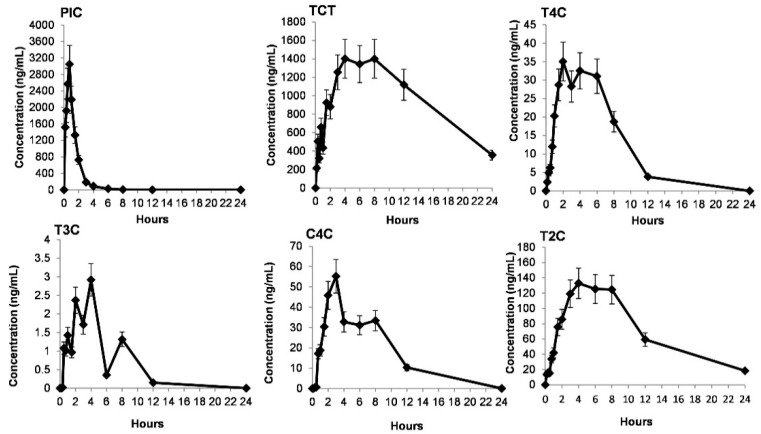
Mean plasma concentration-time curves of six constituents identified and quantified after oral administration of Kashmir saffron extract (CSE) at the dose of 40 mg/kg in rats (mean ± SEM, *n =* 6). PIC, T4C, TCT, T3C, C4C, and T2C.

The method parameters were confirmed and validated for TCT, T4C, PIC, and SAF based on bioanalytical guidelines in the rat plasma. This method was applied to the study of carotenoids and apocarotenoids in plasma after oral administration of CSE in rats. The compounds PIC, SAF T4C, TCT with T3C, C4C, and T2C were determined using the validated UFLC-MS/MS method. The response of compounds T3C, T2C, and C4C were confirmed based on the retention time, relative retention factor from IS1, and optimized MS/MS profile. (Table 2) These compounds were quantified against individual linearity and area response in rat plasma in each analysis [4] (Figure 4)

### 2.4. Pharmacokinetic Results

As is known, natural products may work through the combined effects of constituents with similar structures [44]. Satisfactory therapeutic effects are obtained even though some of the constituents may be at low blood concentrations. The quantification showed a significant amount of T4C, T3C, and PIC with C4C, T2C, TCT, and SAF in CSE by UFLC-PDA. The validated bioanalytical UFLC-MS/MS method was applied to the simultaneous determination of these seven compounds after single oral administration of CSE (40 mg/kg) in rats (*n =* 6). The quantification was done for apocarotenoids PIC and SAF and carotenoids T4C, T3C, C4C, T2C, TCT.

The PK parameters were calculated by non-compartmental analysis. The PK parameters, such as maximum plasma concentration (C*_max_*) and the time to reach the maximum plasma concentration (T*_max_*), were derived directly from the experimental data. The trapezoidal equation calculated the areas under the plasma concentration-time curves from 0 to the time of the last quantifiable concentration (AUC*_0−t_*). The AUC was extrapolated to infinity AUC*_0-∞_* using C*_t_*/K*_el_*, where C*_t_* is the last measured MET concentration. K*_el_* is the elimination rate constant determined from the terminal slope of the log concentration-time plot. The K*_el_* was obtained from the linear regression curve slope by fitting the terminal concentrations’ natural logarithms versus time. The terminal elimination half-life (t*_1/2_*) was calculated by 0.693/K*_el_*. The clearance (CL) was calculated as the quotient of the dose (D) and AUC*_0−∞_.* All values were expressed as mean ± SEM.

As a result, in this study, the crocins detected in plasma were all acquired. This is the first report on the PK profiles of seven compounds of CSE in rats. The PK parameters were calculated and are listed in Table 9. The mean plasma concentration-time curves of six compounds are shown in Figure 4, except SAF due to the low concentration in vivo. The PK parameters of SAF could not be calculated because of the lack of sufficient data points, as its original amount in the CSE was comparatively low.

All six compounds were detected at a first-time point (i.e., 10 min). The TCT reached C*_max_* at 6.83 ± 1.68 h in the plasma, which is higher than the previously reported maximum plasma concentration (C*_max_*), i.e., 66.3 ± 9.2 min in rat plasma [42]. The five CS analytes T4C, T3C, C4C, T2C, and TCT, achieved C*_max_* at between 3 and 7 h, i.e., time to reach the maximum plasma concentration (T*_max_*) except PIC, which attained C*_max_* at 0.67 h in the plasma. The concentration-dependent responses of PIC, T4C, C4C, T2C, T3C, and TCT are shown in Figure 4 except for SAF due to its low abundance and AUC in samples.

This is the first study that reports the PK parameters of the PIC in the rats. As shown in Table 9, the t*_1/2_* of TCT was more than 11 h, suggesting that the compounds’ absorption and elimination rates were slower than most carotenoid ingredients after oral administration of CSE in rats. The slow elimination of components might help to maintain effects. The absorption rate of TCT, which had the highest concentration among all components, was relatively slow, maybe because of transformation from T4C, T3C, C4C, and T2C by intestinal bacteria [46,47], and this validated method was found to be efficient to confirm and quantify these analytes responses in rat plasma samples. This concludes that PIC is also a significant biomarker in vivo and TCT after oral KCS and CSE consumption.

## 3. Discussion

While the latest methodologies and reports are [17,22,29] targeting only T4C study in PK or bioanalysis, this research gives a novel method of quantification and study for five more bioactive compounds from CS ( i.e., T3C, T2C, C4C, PIC, and SAF) for further application in any preclinical or clinical studies. Most of the saffron bioanalytical studies lack the validation data needed for the reproducibility and robustness of these carotenoids and apocarotenoids quantification methods. The current study gives a stable matrix-based extraction and validation of UFLC-MS/MS-based methodology, consistent and sensitive for all these compounds.

Some pharmacokinetic studies on CS reported that the intestinal deglycosylation of various crocins is primarily due to the enzymatic processes in the epithelial cells. In these reports, minor crocins may be deglycosylated by the fecal microbiome, facilitating transformation into TCT finally [36,37]. The values of C*_max_* and AUC*_0−t_* of TCT in the present study were much higher than other crocins. This phenomenon might be the biotransformation of crocins to TCT by intestinal bacteria and enzymes in vivo, leading to a higher concentration of TCT in plasma. The comparative results indicated that coexisting compounds in CS might enhance the absorption of TCT by increasing absorption or bioavailability. However, the exact absorption mechanism of these components is still unclear.

## 4. Materials and Methods

### 4.1. Chemicals and Reagents

Compounds used in this study are *trans*-4-GG-crocin (T4C) (Pubchem CID-24721245), *trans*-3-Gg-crocin (T3C) (Pubchem CID-9940690), *trans*-2-gg-crocin (T2C) (Pubchem ID- 25244294), *cis*-4-GG-crocin (C4C) (Pubchem CID-101662426), *trans* crocetin (TCT) (Pubchem CID-124350893), picrocrocin (PIC) (Pubchem CID-130796), safranal (SAF) (Pubchem CID-61041), reserpine (Pubchem CID-5770), and chloramphenicol (Pubchem CID-5959). The reference compounds were procured from the following suppliers: T4C and T3C procured from Chromadex (Los Angeles, CA, USA) and PhytoLab, (Vestenbergsgreuth, Germany), respectively. SAF, reserpine, and chloramphenicol were procured from Sigma–Aldrich (St. Louis, MO, USA). Both PIC and TCT were isolated in-house using previously reported extraction and column chromatography methods [4], further characterized by nuclear magnetic resonance (NMR), MS/MS, and UHPLC-PDA analysis. Both C4C and T2Cwere received as a gift sample from CSIR-IIIM (Jammu, India) [3,4,48]. The purity of reference compounds was checked by analyzing high-concentration (1 mg/mL) solution by UHPLC-PDA and found (>90.0%). Acetonitrile, methanol, water (JT Baker, India), and formic acid, MS grade (Fluka, Honeywell, India), were used in the UFLC-MS/MS study.

### 4.2. Preparation and Standardization of Kashmir Saffron Extract (CSE)

The Kashmir saffron sample was procured from Jammu and Kashmir, India, (KCS) and authenticated by the Botanical Survey of India, Jodhpur. The dried stigmas of saffron were ground to a coarse powder (200 g) and extracted with ethanol-water at 40 °C for 3 h. Extraction was repeated twice, followed by distillation of solvent below 50 ℃ with yield (20%). The powder obtained after distillation was stored in an amber-colored bottle below 2 °C (CSE). The CSE (110g), suspended in water and then partitioned with hexane-ethyl acetate (5:95). Further from this, hexane-ethyl acetate layer fraction (12 g), PIC (28 mg), and TCT (34 mg) were isolated and further characterized by nuclear magnetic resonance (NMR) and MS/MS analysis [4].

The contents of PIC, SAF, T4C, T3C, C4C, T2C, and TCT, the CSE was standardized and quantified by an external standard method by similar chromatographic conditions as the experimental section by the UFLC-PDA method [49,50,51]. The PIC and SAF’s identification and quantification were recorded at 254 nm and 320 nm against the reference standards. All the isomers of crocins and TCT were identified and quantified at 440 nm against T4C and TCT, respectively. (Figure S1).

### 4.3. Animals

Male Sprague–Dawley rats (250–300 g) were purchased from Crystal Biological Solution, Pune, Maharashtra, India. The animal studies were sanctioned by the Animal Ethics Committee of Crystal Biological Solution, Pune. It was carried out following the Animal Ethics Procedures and Guidelines of Control and Supervision of Experiments on Animals (CPCSEA) committee requirements. Animals were housed 3–4 per cage in rooms with constant temperature (25 ± 2 °C), humidity (50 ± 20%), and 12 h dark-light cycle. All animals in the oral group fasted overnight before the dosing, and food was provided 8 h post-dose, and water was ad libitum. The investigational CSE was fed orally to an individual rat as per its body weight.

### 4.4. Ultrafast Liquid Chromatography-Mass Spectrometry (UFLC-MS/MS)

The UFLC-MS/MS analysis was performed on a Shimadzu UFLC system (Kyoto, Japan) consisting of a DGU-20A5R degasser, LC-30AD pump, SIL-30AC autosampler, CTO-20AC column oven. The chromatographic separation was carried out in a single analytical run divided into loading and eluting phases by alternating the electronic valve. At the loading phase (−1–0 min), the auto-sampler was responsible for injecting 50 µL of the sample. The LC-30AD pump was responsible for delivering water containing 0.1% formic acid to the pre-column (Phenomenex Security Guard ULTRA with C8 Cartridge) at 0.8 mL/min to remove protein and retain analytes. At the eluting phase (0–12 min), the retained components were flushed from the pre-column into the analytical column (Dr. Maisch GmbH Reprosil Gold XBD C8, 50 mm × 4.6 mm, i.d., 1.8 µm) eluting as per time programmed gradient. The flow rate of the mobile phase was 0.8 mL/min. The mobile phase consists of a mixture of water containing 0.1% formic acid (%*v*/*v*) (A) and 100% acetonitrile (B). The gradient elution program was carried out for chromatographic separation: 0.01–1.91 min, 20% B; 1.91–5.91 min, 2090% B; 5.91–6.91 min, 90–80% B; 6.91–8.24 min, 80–20% B; and 8.24–12 min, 20% B. The column oven temperature was set at 30 °C. The MS/MS analysis was carried out on an LCMS-8045 triple-quadrupole mass spectrometer (Shimadzu, Kyoto, Japan) equipped with positive and negative electrospray ionization (ESI) interfaces using the MRM mode. The compound and source dependent parameters were defined as follows: nitrogen was used as nebulizing and drying gas; argon was used as the collision gas. Quadruple voltage was set at Q1 RF gain: 4998 Q1 RF offset 4990 and Q1 post-rod bias: −5.0 V CID CELL exit lens: −4.0 V, interface: ESI, interface temperature: 350 °C, desolvation line (DL) temperature: 250 °C, nebulizing gas flow: 2.50 L/min, heating gas flow: 10.00 L/min heat block: 300 °C; drying gas flow: 10.00 L/min.

The ESI source was operated in the negative and positive ion mode. The MRM analysis was done by monitoring transitions of the precursor to product ions transition of compounds, i.e., *m/z* 975.7/651.3 for T4C, *m/z* 813.3/327.2 for T3C, *m/z* 651.3/327.2 for T2C, *m/z* 975.5/651.4 for C4C, *m/z* 327.1/283.1 for TCT, *m/z* 609.7/195.1 for reserpine, and 321.00/152.00 for chloramphenicol. The IS1 (reserpine) was used to analyze PIC and SAF in positive mode, whereas IS2 (chloramphenicol) was used to analyze T4C, T3C, T2C, C4C, and TCT in a negative mode. The apocarotenoids PIC showed *m/z* 151.3/81.1, similar to the SAF *m/z* 151.3/81.1 but separated by LC based on retention time 1.925 and 6.190 min, respectively (Figure 3). The other instrument parameters, viz. dwell time (msec), Q1 Pre-Bias (eV), CE (eV), Q3 Pre-Bias (eV), tR (min), were optimized as per Table 2. All data were controlled and analyzed by Lab Solution and Lab Solution Insight Software (Versions 3.2) of Shimadzu Tech. (Kyoto, Japan).

### 4.5. Preparation of Standard Solution, Calibration Standards, and Quality Control Samples

Each standard stock solution of T4C, T3C, T2C, C4C, TCT, PIC, and SAF (1.0 mg/mL) with IS1and IS2 (1.0 mg/mL) was prepared separately in the methanol to prepare calibration standards and quality control samples. The primary stock solutions were made up of methanol to prepare mixed working solutions (WSa). All the standard stock solutions were stored at 4 °C. The calibration curve (CC) stock solutions (WSa) were diluted with methanol to prepared working solutions with a range of 100 to 32,000 ng/mL (WSb). The stock solutions of IS1 and IS2 were diluted to prepare 0.1 mg/mL (WSc).

The blank plasma samples of volume 45 µL were spiked with each of 5 µL standards (WSa) and IS (WSc) solutions, respectively. With further 200 µL, methanol was added and vortex for 2 min, followed by centrifugation for 10 min at 2000 RPM. The supernatant was collected in HPLC vials for analysis. A nine-point CC (10, 30, 100, 200, 400, 800, 1600, 2400, and 3200 ng/mL) was prepared for analysis. Quality control samples were prepared separately by spiking respective working standard solutions to achieved LLOQ (10 ng/mL), LOQ (30 ng/mL), MOQ (1600 ng/mL), and HOQ (2400 ng/mL).

#### 4.5.1. Preparation of Plasma Samples

An aliquot of 45 µL of rat plasma was spiked with each 5 µL of IS1 and IS2, and 200 µL of methanol was added, vortexed for 1–2 min, then centrifuged for 10 min at 2000 RPM. Collect the supernatant into HPLC vials and injected it into the UFLC-MS/MS system for analysis.

#### 4.5.2. Linearity and Calibration Curve (CC)

The linearity of T4C, T3C, T2C, C4C TCT, PIC, and SAF over nine points (10, 30, 100, 200, 400, 800, 1600, 2400, and 3200 ng/mL) were prepared by spiking of 45 µL drug-free rat plasma with the appropriate amount of analyte and IS1 and IS2. The linearity was assessed with three different calibration curves with maintaining the standard internal concentration at 10 ng/mL. A nine-point CC was set up by plotting T4C, T3C, T2C, C4C, TCT, PIC, and SAF peak area ratio against the control matrix’s nominal concentration of calibration standards. Calculation of linear regression data with 1/X^2^ weighting factor.

### 4.6. Bioanalytical Validation: UFLC-MS/MS Method

The optimized method for carotenoids T4C, T3C, C4C, T2C, TCT, and apocarotenoids PIC and SAF were further validated for four bioactive compounds, i.e., TCT, T4C, PIC, and SAF based on the reference compound’s availability. The compounds T3C, T2C, and C4C were confirmed based on the retention time, relative retention factor from IS, and MS/MS profile [3].

#### 4.6.1. System Suitability Test

A system suitability test (SST) was performed before the batch analysis. The LLQC (*n =* 7) sample was followed by a blank (*n =* 8) injected in the system to assess the reproducibility of tR and peak area response in the method. Acceptance criteria for the relative standard deviation (RSD) of the analyte peaks’ area response obtained from the seven-system suitability; plasma samples were <10.00%.

#### 4.6.2. Specificity, Selectivity, and Sensitivity (LLOQ)

The method’s specificity and selectivity were determined by analyzing samples from at least six different blank rat plasma sources for significance interference at the LC peak elution zone of T4C, TCT, PIC, SAF, and IS. Further plasma samples spiked with IS1 and IS2 were analyzed as zero calibrators and spiked with mixed analyte at LLOQ level for any co-eluting peak interference at the LC peak elution zone. Sensitivity was evaluated at the LLOQ with acceptable precision, accuracy, and signal-to-noise (S/N) ratio was >10.

#### 4.6.3. Quality Control Samples

After determining the linearity of T4C, TCT, PIC, and SAF nine points calibration curve (in Section 4.5.2), four QC levels were calculated [41]. The QC samples were prepared at four levels by spiking the analytes in plasma to get the LLOQ (lower limit of specification), LQC (three times of LLOQ), MQC (50% of the upper CC range), and HQC (75% of the upper CC range).

#### 4.6.4. Accuracy and Precision

The intraday accuracy and precision were ascertained by analyzing QC samples spiked at four levels within the calibration curve (10 ng/mL, 30 ng/mL, 1600 ng/mL, 2400 ng/mL) in blank rat plasma. Intraday precision and accuracy of the method were estimated in multiple analyses of batches (*n =* 6) of quality control samples for repeatability. And for the inter-day analysis, the same sample set was analyzed on three consecutive days. The acceptance criterion for each back-calculated standard concentration was 85–115% accuracy from the nominal value, except for the LLOQ (<80–120%). The precision criterion was <15% RSD (Table 3 and Table 4) except for the LLOQ (<20%).

#### 4.6.5. Extraction Recovery, Matrix Effect, and Carryover

Analyte recovery was calculated at three quality control levels in blank plasma. QC samples were prepared (*n =* 6) at the low, medium, and higher QC (30, 1600, 2400 ng/mL), by spiking freshly prepared mix analyte and IS (1.0 µg/mL) into the blank matrix. Following extraction, recovery (%) was determined by comparing QC samples’ mean response at pre-extraction spiking with corresponding post-extraction spiking. The matrix effect was demonstrated as a matrix factor at two quality control levels in blank plasma. The QC samples (LQC and HQC) were prepared (*n =* 6), containing the analyte and IS spiked into a blank plasma matrix (post-extraction samples). The other containing Analyte and IS spiked into the mobile phase (diluent). The matrix factor was evaluated as the ratio of peak response of analyte and IS in the presence of matrix divided by IS and analyte’s peak response in the absence of matrix. The carryover was studied by comparing the response in a blank after calibration standard at the ULOQ (*n =* 3). The total response was noted and monitored for not exceeding 20% of LLOQ.

#### 4.6.6. Stability Studies of Carotenoids and Apocarotenoids

The stability was performed at different sets of working and storage conditions. As carotenoids and apocarotenoids are sensitive compounds, their presence in CSE was studied carefully. The degradation of T4C, TCT, PIC, and SAF was observed in plasma at LQC and HQC levels. The stability conditions of the method studied were at ambient temperature for 4 h (room-temperature stability), in the autosampler at 8 °C conditions for 24 h (autosampler stability), and with three freeze-thaw cycles at −20 ± 4 °C condition (freeze-thaw stability). The samples were prudent to be stable if the measured concentration was within ±15% of the nominal concentration [40,41].

### 4.7. Application of Validated Methodology in Investigation of Pharmacokinetic Parameters of CSE

This sensitive and validated UFLC-MS/MS method was applied for the simultaneous determination of CSE apocarotenoids and carotenoids in rat plasma. The PK parameters were investigated after oral administration of CSE in the rats (*n =* 6). The in vivo pharmacokinetic study of KCS was studied for active metabolite TCT and T4C with PIC, SAF with T3C, C4C, and T2C.

### 4.8. Pharmacokinetic Study

Twelve male animals were free to access the water and food until 12 h before the experiment. In the study, the animals were administered 40 mg/kg CSE by oral gavage. The dose was selected based on previous research [3,4,52]. After drug administration, twelve animals were further subdivided into two groups with six animals (for alternate time-point blood samples). Blood samples (0.2 mL) were collected from the ophthalmic venous plexus into heparinized tubes at pre-dose, 0.16, 0.33, 0.5, 0.75, 1, 1.5, 2, 3, 4, 6, 8, 12, and 24 h post-dosing, respectively. Heparin sodium (1 g/100 mL) with 10 µL was added to a tube and dried into the heparinized tube. In the experiment, the saline and sugar solutions were provided every two h to promote the recovery of the rat’s blood volume. The blood samples were collected from six rats at each time point. Each blood sample was centrifuged at 5000 rpm for 10 min, and then the plasma layer was transferred into clean tubes and stored at −80 °C until analysis.

## 5. Conclusions

India’s Kashmir region has been found suitable for the growth and quality supply of KCS with regional agricultural benefit. The KCS and its extract (CSE) has become a popular dietary supplement with bioactive as apocarotenoids (PIC, SAF) and carotenoids (crocins, crocetin) [3,28,29,30,31,32,33,34]. These dietary supplements have been regulated widely and require a validated methodology and data. [53,54]. The studies showed the potential of KCS and CSE in brain health. Therefore, the quantitative relationship of apocarotenoids, carotenoids, and active metabolites like crocetin in the CSE and it’s in vivo therapeutics data hold importance showed an improved memory function.

Thus, in this research, a sensitive and reproducible UFLC-MS/MS method was developed for the simultaneous determination of seven CSE compounds in rat plasma. The method showed excellent linearity (*R*^2^ > 0.990) with the lower limit of quantification (LLOQ) (10 ng/mL) for apocarotenoids PIC and SAF and carotenoids T4C, T3C, C4C, T2C, TCT.

This UFLC-MS/MS method was validated to determine PIC, SAF TCT, and T4C with internal standards reserpine and chloramphenicol in the rat plasma. The precision (RSD, <15%) and accuracy (RE, −11.03–9.96) studies on UFLC-MS/MS assay based on the three analytical quality control (QC) levels were well within the acceptance criteria from FDA guidance for bioanalytical method validation with recoveries (91.18–106.86%) [40]. The method was applied to investigate the PK parameters after oral administration of 40 mg/kg CSE in the rats (*n =* 6).

This is the first report on the in vivo oral pharmacokinetics investigations that disclosed active metabolite PIC with TCT, T4C quantitatively related to CSE. The T3C, C4C, T2C, and SAF were also detected by this validated bioanalytical method after oral CSE consumption. This will help preclinical/clinical trials of KCS dietary supplements focusing on apocarotenoids and carotenoids in various therapeutic applications and their neuroprotective role.

## 6. Patents

Sustained-release formulations of Crocus sativus. IN201711036084, WO2019077621A1, EP18796784.9, US16753969.

## Figures and Tables

**Table 1:** Quantitation of Kashmir saffron extract (CSE) (*n =* 3) (%, *w*/*w*) for compounds PIC, SAF, T4C, T3C, C4C, T2C and TCT.

Analytes	T4C	T3C	T2C	C4C	TCT	SAF	PIC
CSE	13.76 ± 0.280	5.50 ± 0.418	0.875 ± 0.0255	0.624 ± 0.011	0.0378 ± 0.0015	0.033 ± 0.00050	18.09 ± 0.586

**Table 2:** Precursor/product ion pairs and parameters for multiple reaction monitoring (MRM) of compounds used in this study.

Analyte	Formula	MW (g/mol)	Ionization Mode	Precursor Ion (*m*/*z*)	Product ion (*m*/*z*)	Dwell Time (msec)	Q1 Pre-Bias (eV)	CE (eV)	Q3 Pre-Bias (eV)	Retention Time, tR (min)
T4C	C_44_H_64_O_24_	976.38	-ve	975.70	651.25	53.00	22.00	23.00	34.00	3.90
					327.20	53.00	28.00	37.00	22.00	
T3C	C_38_H_54_O_19_	814.33	-ve	813.30	327.20	53.00	18.00	28.00	16.00	4.12
					651.20	53.00	18.00	14.00	18.00	
T2C	C_32_H_44_O_14_	652.27	-ve	651.25	327.15	53.00	14.00	20.00	16.00	4.74
					283.20	53.00	22.00	26.00	32.00	
C4C	C_44_H_64_O_24_	976.38	-ve	975.70	651.25	53.00	22.00	23.00	34.00	4.53
					327.20	53.00	22.00	38.00	15.00	
TCT	C_20_H_24_O_4_	328.17	-ve	327.10	283.10	53.00	12.00	9.00	13.00	5.96
					239.35	53.00	12.00	12.00	26.00	
PIC	C_16_H_26_O_7_	330.17	+ve	151.25	81.05	62.00	−16.00	−22.00	−14.00	1.93
					123.15	62.00	−10.00	−17.00	−24.00	
SAF	C_10_H_14_O	150.10	+ve	151.25	81.05	53.00	−16.00	−22.00	−14.00	6.19
					123.15	53.00	−10.00	−17.00	−24.00	
IS1	C_33_H_40_N_2_O_9_	608.27	+ve	609.70	195.10	53.00	−22.00	−40.00	−20.00	4.82
					448.05	53.00	−22.00	−31.00	−22.00	
IS2	C_11_H_12_Cl_2_N_2_O_5_	322.01	-ve	321.00	152.00	53.00	16.00	11.00	18.00	4.42
					257.00	53.00	11.00	16.00	15.00	

T4C—*trans*-4-gg-crocin; T3C—*trans*-3Gg-crocin; T2C—*trans*-2gg-crocin; C4C—*cis*-4GG-crocin; TCT—*trans* crocetin; PIC—picrocrocin; SAF—safranal; IS1—internal standard 1; IS2—internal standard 2.

**Table 3:** The linear equation, linear range, and LLOQ of the T4C, T3C, T2C, C4C, TCT, PIC, and SAF in rat plasma samples.

Analyte	Linear Equation	Range (ng/mL)	*R* ^2^	LOD (ng/mL)	LLOQ (ng/mL)
T4C	10.00 to 3200.00	y = 0.001106907x + 0.0007873082	0.9968	2.00	10.00
T3C	10.00 to 3200.00	y = 0.002373485x − 0.000003492705	0.9954	2.00	10.00
T2C	10.00 to 3200.00	y = 0.002490268x − 0.0001086531	0.9990	5.00	10.00
C4C	10.00 to 3200.00	y = 0.0002475676x + 0.0003535316	0.9960	5.00	10.00
TCT	10.00 to 3200.00	y = 0.002001102x + 0.002474666	0.9952	2.00	10.00
PIC	10.00 to 3200.00	y = 0.002674921x + 0.004357420	0.9975	5.00	10.00
SAF	10.00 to 3200.00	y= 0.007429112x + 0.02994744	0.9985	2.00	10.00

**Table 4:** Intra- and inter-day precision and accuracies of the analytes in rat plasma, (ng/mL).

Analyte	Nominal Concentration (ng/mL)	Intra-Day 01 (*n =* 6)		Intra-Day 02 (*n =* 6)		Intra-Day 03 (*n =* 6)		Intra-Day 04 (*n =* 6)		Inter-Day (*n =* 6 × 3)	
		Precision (RSD, %)	Accuracy (RE, %)	Precision (RSD, %)	Accuracy (RE, %)	Precision (RSD, %)	Accuracy (RE, %)	Precision (RSD, %)	Accuracy (RE, %)	Precision (RSD, %)	Accuracy (RE, %)
T4C	10.00	6.76	−2.78	6.76	−2.78	7.96	−1.09	5.72	−6.03	6.93	5.53
	30.00	6.65	−2.33	6.65	−2.33	6.65	−3.97	2.98	−1.95	5.53	−7.62
	1600.00	1.23	−2.73	1.23	−2.73	3.43	−6.39	2.92	−7.50	1.16	−8.92
	2400.00	5.15	−0.19	5.15	−0.19	5.48	−1.94	2.25	−7.77	1.69	2.15
TCT	10.00	7.40	0.92	7.40	0.92	7.18	−1.16	6.97	−5.52	6.07	1.12
	30.00	4.95	−0.63	4.95	−0.63	5.67	−3.12	6.31	−3.91	5.74	−4.83
	1600.00	1.98	2.29	1.98	2.29	5.63	−4.44	1.29	−7.14	3.49	−8.48
	2400.00	5.81	1.68	5.82	1.68	4.38	−1.21	1.94	−3.99	2.48	−1.32
PIC	10.00	7.04	−11.03	7.04	−11.03	9.96	−3.71	6.70	0.25	5.24	4.63
	30.00	5.95	−6.23	5.95	−6.23	8.00	−3.20	4.83	−0.77	10.51	−5.14
	1600.00	2.50	−1.50	2.50	−1.50	3.99	−5.72	2.90	−3.97	2.08	−8.32
	2400.00	1.57	0.24	1.57	0.24	3.35	−1.83	4.09	−4.98	3.34	−0.61
SAF	10.00	5.11	4.90	5.11	4.90	6.61	3.83	10.18	−4.73	7.41	6.35
	30.00	5.15	−1.26	5.15	−1.26	5.45	−1.18	5.21	1.77	7.14	−1.49
	1600.00	2.24	−2.95	2.24	−2.95	3.21	−4.85	2.59	−7.35	2.33	−7.63
	2400.00	2.91	−0.20	2.91	−0.20	3.88	−1.51	1.96	−5.11	1.95	0.66

**Table 5:** Extraction recovery(ng/mL) of the compounds in rat plasma samples, (*n =* 6).

Analyte	Nominal Concentration (ng/mL)	Extraction Recovery (%, mean ± SD)	Precision (RSD, %)	Accuracy (RE, %)
T4C	30.00	91.18 ± 0.73	0.80	−9.29
	1600.00	99.09 ± 1.55	1.56	−1.15
	2400.00	101.60 ± 0.46	0.45	1.56
TCT	30.00	106.23 ± 3.62	3.41	5.99
	1600.00	105.22 ± 3.48	3.30	4.45
	2400.00	101.45 ± 1.01	1.00	−2.18
PIC	30.00	96.74 ± 1.04	1.07	−4.30
	1600.00	102.89 ± 1.76	1.71	2.04
	2400.00	106.86 ± 1.73	1.61	4.67
SAF	30.00	100.72 ± 0.58	0.57	0.91
	1600.00	103.05 ± 2.95	2.86	2.38
	2400.00	105.29 ± 1.83	1.74	4.98

**Table 6:** Dilution Integrity of analytes in two-fold and four-fold dilution (ng/mL) (*n =* 6).

Analyte	Nominal Concentration (ng/mL)	Two-Fold Dilution (ng/mL)	Precision (RSD, %)	Accuracy (RE, %)	Four-Fold Dilution (ng/mL)	Precision (RSD, %)	Accuracy (RE, %)
T4C	5000.00	4663.09 ± 92.81	1.99	−6.74	4642.16 ± 118.87	2.56	−7.16
TCT		4634.89 ± 137.40	2.96	−7.30	4689.90 ± 182.80	3.90	−6.20
PIC		4740.06 ± 164.85	3.48	−5.20	4744.10 ± 118.81	2.50	−5.12
SAF		4706.07 ± 57.69	1.23	−5.88	4722.58 ± 62.50	1.32	−5.55

**Table 7:** Matrix effect (ng/mL) for the determination of compounds in rat plasma (*n =* 6).

Analyte	Nominal Concentration (ng/mL)	Matrix Effect (%, mean ± SD)	Precision (RSD, %)	Accuracy (RE, %)
T4C	30.00	100.03 ± 5.21	5.21	−7.63
	2400.00	98.98 ± 4.17	4.21	3.33
TCT	30.00	97.68 ± 8.35	8.55	−2.25
	2400.00	100.78 ± 2.59	2.57	−2.08
PIC	30.00	95.88 ± 8.44	8.81	2.14
	2400.00	98.91 ± 6.91	6.99	0.71
SAF	30.00	96.43 ± 6.51	6.75	2.18
	2400.00	99.19 ± 2.53	2.55	1.51

**Table 8:** Stability assays for the determination of compounds in rat plasma (*n =* 6).

Analyte	Nominal Concentration (ng/mL)	Autosampler Stability (%, mean ± SD)	Precision (RSD, %)	Accuracy (RE, %)	Room Temperature Stability (%, mean ± SD)	Precision (RSD, %)	Accuracy (RE, %)	Freeze-Thaw Stability (%, mean ± SD)	Precision (RSD, %)	Accuracy (RE, %)
T4C	30.00	29.01 ± 1.38	4.76	−3.29	27.80 ± 1.29	4.64	−7.34	28.45 ± 1.06	3.74	−5.18
	2400.00	2481.58 ± 86.48	3.49	3.40	2413.86 ± 73.67	3.05	0.58	2369.47 ± 195.39	8.25	−1.27
TCT	30.00	29.94 ± 1.29	4.30	−0.19	28.59 ± 1.18	4.12	−4.69	29.79 ± 1.58	5.30	−0.69
	2400.00	2467.92 ± 101.06	4.09	2.83	2425.78 ± 112.27	4.63	1.07	2383.90 ± 133.23	5.59	−0.67
PIC	30.00	29.52 ± 1.26	4.26	−1.61	30.40 ± 0.92	3.01	1.33	29.98 ± 1.66	5.55	−0.06
	2400.00	2463.69 ± 113.42	4.60	2.65	2406.19 ± 94.01	3.91	−0.26	2498.53 ± 111.53	4.46	4.11
SAF	30.00	29.97 ± 0.90	3.08	−2.44	28.07 ± 0.67	2.38	−6.43	29.66 ± 1.42	4.78	−1.13
	2400.00	2472.88 ± 0.90	4.08	3.04	2370.67 ± 126.71	5.35	−1.22	2499.17 ± 60.75	2.43	4.13

**Table 9:** Pharmacokinetics (PK) parameters of six compounds after an oral administration of Kashmir saffron extract (CSE) (*n =* 6).

PK Parameters	T4C	T3C	T2C	C4C	TCT	PIC
C*_max_* (ng/mL)	49.27 ± 11.15	7.59 ± 4.71	160.44 ± 15.17	86.14 ± 15.65	2076.21 ± 373.61	2722.95 ± 231.41
T*_max_* (h)	3.5 ± 0.57	3.5 ± 1.09	4.34 ± 0.51	3.25 ± 1.05	6.84 ± 1.69	0.792 ± 0.10
AUC_0-*t*_ (h.ng/mL)	277.04 ± 67.69	20.36 ± 9.64	1529.1 ± 197.40	395.64 ± 113.41	23,590 ± 3119.25	3691.19 ± 274.38
AUC_0-∞_ (h.ng/mL)	370.45 ± 75.64	55.46 ± 31.21	1676.12 ± 238.38	407.21 ± 187.28	30,679.13 ± 3706.46	3818.76 ± 256.67
t*_1/2_* (h)	3.36 ± 1.00	4.6 ± 2.74	5.75 ± 0.73	1.57 ± 1.57	8.98 ± 2.00	0.793 ± 0.078
CL (mL/h/kg)	19,568.35 ± 4835.41	114,958.33 ± 71,000.46	234.77 ± 31.12	326.37 ± 445.89	0.547 ± 0.094	1935.88 ± 123.45
V*_d_* (L/kg)	69.250 ± 16.150	325.573 ± 13.099	2.006 ± 0.457	4.29 ± 1.94	0.00617 ± 0.00104	2.258 ± 0.3168

C*_max_*- maximal observed concentration; T*_max_*- maximum observed time; AUC*_0-t_*- area under the curve from time zero to the last measurable concentration; AUC*_0-∞_*- area under the curve from time zero extrapolated to infinite time; t*_1/2_* - half-life; CL- clearance; V*_d_*-volume of distribution; h-hour; ng-nanogram; mL-milliliter; L-liter; kg-kilogram.

## Supplementary Materials

The following are available online. Figure S1: HPLC Standardization of *Crocus sativus* extract A- Chromatograms of standards PIC, T4C, TCT, SAF; B- Chromatograms of KCS extract at 254 nm/440 nm/320 nm.

## Sample Availability

Samples of the compounds are available from the authors.
