# Supplementary material for: Bioanalytical Method Development and Validation Study of Neuroprotective Extract of Kashmiri Saffron Using Ultra-Fast Liquid Chromatography-Tandem Mass Spectrometry (UFLC-MS/MS): In Vivo Pharmacokinetics of Apocarotenoids and Carotenoids
Source: Molecules. 2021 Mar 23;26(6):1815. doi: 10.3390/molecules26061815 (PMC8005090; doi:10.3390/molecules26061815)

Supplementary information

Bioanalytical Method Development and Validation Study of Neuroprotective extract of Kashmiri Saffron using Ultra-Fast Liquid Chromatography-Tandem Mass Spectrometry (UFLC-MS/MS): In-vivo Pharmacokinetics of Apocarotenoids and Carotenoids

Aboli Girme*, Sandeep Pawar, Chetana Ghule, Sushant Shengule, Ganesh Saste, Arun Kumar Balasubramaniam, Amol Deshmukh, Lal Hingorani

Pharmanza Herbal Pvt. Ltd., Anand, Gujarat, India.

***Corresponding author**

Aboli Girme, Pharmanza Herbal Pvt. Ltd.

Present/ permanent address: Plot # 214, Borsad-Tarapur Road, Nr. Vadadla Patiya, At & PO: Kaniya-388435, Ta: Petlad, Dist: Anand (Gujarat) India.

Tel.: +91 7043534016, +91 9825063959

E-mail address: ardm@pharmanzaherbals.com (Aboli Girme)

Figure S1: UFLC-PDA Standardization of Kashmir Saffron (*Crocus sativus*) extract (CSE) **A**- Chromatograms of reference standards PIC, T4C, TCT, SAF; **B**- Chromatograms of CSE sample at 254 nm/440 nm/320nm.

Figure S1: UFLC-PDA Standardization of Kashmir Saffron (*Crocus sativus*) extract (CSE) **A-** Chromatograms of reference standards PIC, T4C, TCT, SAF; **B-** Chromatograms of CSE sample at 254 nm/440 nm/320nm.

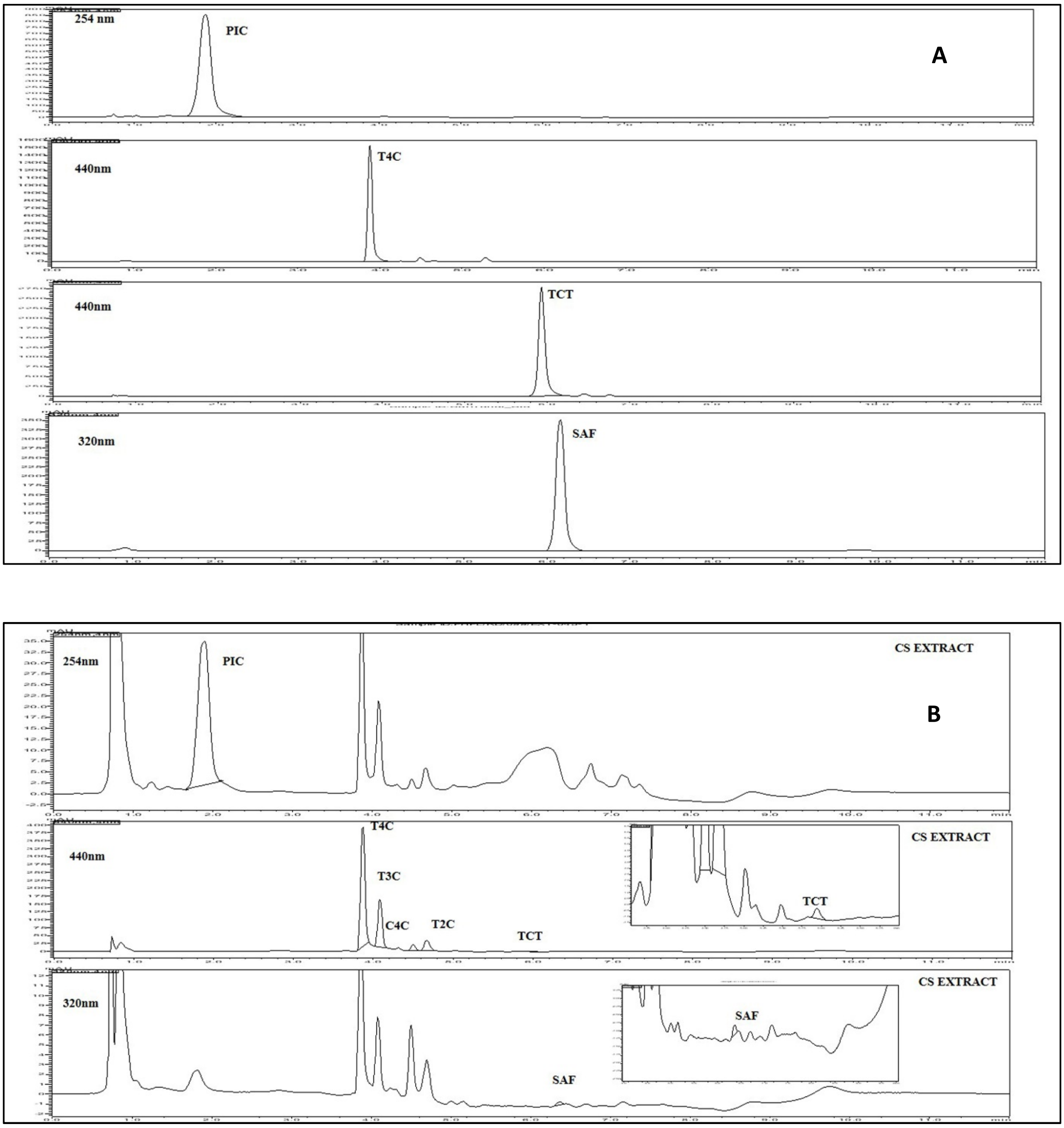

Supplement: Supplementary file 1 [file molecules-26-01815-s001.pdf]
